# Topic based quality indexes assessment through sentiment

**DOI:** 10.1007/s00180-022-01284-7

**Published:** 2022-09-20

**Authors:** Marco Ortu, Luca Frigau, Giulia Contu

**Affiliations:** Department of Economics and Business Sciences, University of Cagliari, Viale Fra Ignazio 17, Cagliari, 09123 Italy

**Keywords:** Multivariate analysis, Partial least squares, Complex analysis, Student satisfaction, University

## Abstract

This paper proposes a new methodology called TOpic modeling Based Index Assessment through Sentiment (TOBIAS). This method aims at modeling the effects of the topics, moods, and sentiments of the comments describing a phenomenon upon its overall rating. TOBIAS is built combining different techniques and methodologies. Firstly, Sentiment Analysis identifies sentiments, emotions, and moods, and Topic Modeling finds the main relevant topics inside comments. Then, Partial Least Square Path Modeling estimates how they affect an overall rating that summarizes the performance of the analyzed phenomenon. We carried out TOBIAS on a real case study on the university courses’ quality evaluated by the University of Cagliari (Italy) students. We found TOBIAS able to provide interpretable results on the impact of discussed topics by students with their expressed sentiments, emotions, and moods and with the overall rating.

## Introduction

Different researchers in time have investigated universities and their activities. They have analyzed aspects as the scientific research and its impact in different fields (see for instance Wang and Wang (2021); Wang et al. (2022)); the programs of international mobility of the university students (see for instance Contu et al. (2020); Amendola and Restaino (2017)); the teaching activities (see for instance Becher (2022)) and the quality of the services and the students’ satisfaction (see for instance Trisihnyo and Harun (2021)). Particularly important to improving education quality are the studies related to evaluating academic teaching activity and of university courses (Park and Cheong 2018; Guo and Yu 2020). They represent constructive feedback that could enhance the future student learning outcomes (Baddam et al. 2019). During the time, it has become an essential component of an education management system (Rajput et al. 2016).

Universities have defined tools able to collect information about the needs of students through which improving their satisfaction and the sentiment felt in attending the courses (Park and Cheong 2018). All this information are then elaborated by specific tools for defining overall ratings of courses and/or lecturers’ effectiveness (Nikolaidis and Dimitriadis 2014). Generally, the student evaluations were carried out at the end of the semester using paper-based surveys. Recently, paper formats have been replaced by online ones (Baddam et al. 2019) together with new instruments to express positive, negative or neutral sentiments (Shen and Zhang 2018) such as e-mails, chat messages, digital notes, and online discussions (Okoye et al. 2020).

In literature, different methodologies have been carried out and introduced to evaluate the effectiveness of lecturers and their courses, such as text mining, machine learning, natural language processing, statistics, corpus linguistics, and visualization techniques (Guo and Yu 2020; Shen and Zhang 2018). Beyond them, other two methodologies are arising in the field of the evaluation of lecturers and university courses: for categorizing students’ comments into positive, neutral, or negative (i.e. Sentiment Analysis) and for identifying the areas of problems (Topic Modeling). Partial Least Square Models have been used by Law and Fong (2020) in the context of academic evaluation. The two authors have used the PLS Structural Equation Modelling (PLS-SEM) to investigate the undergraduate students’ learning transfer. They have proposed a new PLS-SEM approach to analyse learning transfer in English for Academic Purposes (EAP) contexts and to provide insights into the interactivity among four constructs affecting learning (content relevance, understanding of learning, transfer/applicability and transfer outcome) and transfer.

In this paper, we propose a new methodology called TOpic modeling Based Index Assessment through Sentiment (TOBIAS) for inferring the effects of the topics, moods and sentiments of the students expressed in their comments on lecturers and courses upon their overall rating. That is built by combining different techniques and methodologies. Firstly, Sentiment Analysis identifies sentiments, emotions, and moods, whilst Topic Modeling finds the main relevant topics inside comments. Then, Partial Least Square Path Modeling estimates how topics, moods, emotions and sentiments affect the overall rating that summarizes the performance of the analyzed phenomenon.

Four sections, besides the introduction, complete this study. Sect. refsec:relatedspsworks reports the background of the methodologies included in our proposal and the related works. The methodological framework of TOBIAS has been illustrated in Sect. 3. Section 4 shows a motivating example of TOBIAS application, in a real data contest concerning the modeling of students’ evaluation of the lecturers and courses of the University of Cagliari (Italy). Finally, Sect. 5 ends the paper with some concluding remarks.

## Background and related works

### Sentiment analysis

Sentiment analysis (SA) has been defined as a process to extract and process textual data automatically to obtain sentiment information contained in an opinion (Rahmadan et al. 2020). It aims to investigate, analyse, and extract subjective humans’ opinions and sentiments in order to quantify the affective states and subjective information expressed by humans in textual form.

More in detail, SA attempts to detect firstly the *Subjectivity/Objectivity*, which is related to the identification of subjective versus objective text; secondly, the *Polarity*, which aims to assign a qualitative (positive/negative) or quantitative (a number in a given range) sentiment score to a given text; thirdly, the *Discrete Emotions*, which is considered as a more refined grain analysis to extract emotions such as *joy* and *love* from human language.

SA can be applied to different levels of text as document, sentence, and aspect level and different approaches can be used to conduct the analysis (Kaur et al. 2017; Liu 2012). For instance, one is the lexicon-based approach. It can be distinguished in *Dictionary based*, which involves the use of a dictionary of terms built by linguistics experts, who assign a score relative to the sentiment of every single term; and in *Corpus based* approaches, which relies on co-occurrence statistics or syntactic patterns in text corpora and a set of predefined positive and negative seed words (Darwich et al. 2019). Another is the machine learning-based approach, which can be distinguished into three main categories (Madhoushi et al. 2015): the supervised, the unsupervised, and the semi-supervised approaches. *Supervised learning* is a robust and effective solution in traditional document classification, and it is adopted for sentiment analysis with good results (Sodanil 2016). *Unsupervised learning* methods in sentiment analysis do not need prior information in the training data to detect sentiment polarity. An example of such methods is the *rule-based* classifiers (Vashishtha and Susan 2019; Hu et al. 2013). Finally, *semi-supervised learning* learns from both labelled and unlabelled data (Liu 2012). While unlabelled data does not give information about classes, it does provide information on joint distribution over classification features, which is the basic idea driving semi-supervised learning.

Sentiment analysis has been used in many fields and, in time, has become essential to extract information crucial in activities such as *decision-making support*, *business applications* and *predictions and trend analysis*.

Different researchers have used the Sentiment analysis to investigate the sentiment of the students in evaluating lecturers and courses. For instance, Kumar and Jain (2018) have proposed Faculty Evaluation System to evaluate the sentiment evaluation system of a lecturer’s effectiveness to the satisfaction of every student. They have estimated sentiment scores for every feature of a lecturer in order to classify them into three categories good, satisfactory, and unsatisfactory. Wen et al. (2014) have used the SA to investigate the drop out behaviour in three Massive Open Online Course (MOOC). They have discovered the existence of a significant correlation between sentiment expressed in the course forum posts and the number of students who drop the course. Similarly, Wang et al. (2021) have applied the Sentiment analysis to explore the student feelings relate to 18 courses from the Class Central Top 20 MOOCs. They have investigated to analyse the potential connections between instructional design quality and student reviews. Finally, Mujahid et al. (2021) have investigated the effectiveness of e-learning during COVID-19. They have applied sentiment analysis on the Twitter data in order to analyse the polarity and subjectivity score of tweets’ text.

### Topic modelling

Topic modelling (TM) is defined as a mechanism for discovering low-dimensional, multi-faceted summaries of documents or other discrete data Wang and McCallum (2006). It is a process to analyse the words of a text to find hidden themes and the relationship between one theme to another by using statistical methods (Rahmadan et al. 2020). It is an unsupervised learning method because it does not require a document labelling process (Blei 2012).

As evidenced before, different approaches to topic modelling can be used. Among them, LDA is one of the most used because it allows working with extensive collections of text documents (Campbell et al. 2015). LDA is a generative probabilistic model-based introduced by Blei et al. (2003) widely used in several contests such as in the organization of the conferences’ program (Frigau et al. 2021) and computational advertising (Soriano et al. 2013). It is based on the idea that the documents are composed by random mixtures over latent topics, where each topic is characterized by a distribution over words (Jelodar et al. 2019). It operates taking into account three specific elements (Blei et al. 2003): the words, the documents and the corpus. The words are defined as items from a vocabulary indexed by $\{1, \dots ,V \}$ and they represent through a unit-basis vectors. The document is a sequence of *N* words and it is denoted by ${{\textbf {w}}} = (w_{1}, w_{w}, \dots , w_{N})$. The corpus is collection of *M* documents denoted by $D = \{{{\textbf {d}}}_{1}, {{\textbf {d}}}_{d}, \dots , {{\textbf {d}}}_{M}\}$.

The LDA assigns an individual probability, to be generated, to each word. The probability that a specific topic ${{\textbf {t}}}$ generated a word *w* is equal to $\phi _{{{\textbf {t}}},w}$. LDA assumes that $\phi _{{{\textbf {t}}},w}$ is generated by random variable with a symmetric Dirichlet distribution, characterized for a parameter $\beta$.

Moreover, the probability that a document can generate a word from a specific topic is equal to $\gamma _{{{\textbf {w}}},d}$. Its value is originated by a Dirichlet distribution with parameter $\alpha$. When the Dirichlet distribution modelled by the $\alpha$ is supposed symmetric (which is the common assumption), higher values of $\alpha$ implies documents with higher number of topics while lower $\alpha$ implies documents with fewer topics. LDA involves allocating and re-allocating the parameters in $\phi$ and $\gamma$ until the lower bound of the total probability of observing the input documents are maximized.

The parameters are estimated using different methods such as Expectation-maximization (EM), Variational Bayes inference (VB), and Gibbs sampling (Jelodar et al. 2019; Rahmadan et al. 2020). Moreover, different LDA models have been developed as for instance supervised LDA, discriminative LDA, max-entropy discrimination LD, and multimodal LDA (Miao et al. 2021).

In this paper, we use the seeded LDA, a semi-supervised approach. The model uses a weak supervised signal in the form of aspect-specific seed words to identify the topic inside the documents (Lu et al. 2011). In this way, it is possible to identify more coherent aspect-specific topics, while also allowing us to utilize large-scale unlabelled data.

Different researchers have used the LDA to investigate the sentiment of the students in the evaluation of professors and courses. For instance, Cunningham-Nelson et al. (2019) have used the LDA to identify aspects of student opinion of a course. They have applied this model on a dataset containing the satisfaction feedback of students enrolled in the Queensland University of Technology. Hujala et al. (2020) have investigated the sentiment of students enrolled in a Finnish University during two academic years (2016/2017 and 2017/2018). They have attempted to define a process for tapping into the resource of responses to open-ended feedback questions, and they have used LDA to extract topics from written student feedback.

### Partial least squares

Partial Least Squares, first proposed by Wold (1966, 1995), relates the information observed in two sets of tables of observations of the same phenomenon. They are used specifically for : (i) *Correlation*, (ii) *Regression* and (iii) *Path Modeling* (Abdi and Williams 2013). The main tool of PLS is the Single Value Decomposition (SVD), given a $J \times K$ matrix ${\mathbf {Z}}$ (Henseler et al. 2009):

1 $$\begin{aligned} {\mathbf {Z}} = {\mathbf {U}} \Lambda {\mathbf {V}}^{T} = \sum _{l}^{L}\delta _{l}{\mathbf {u}}_{l}{\mathbf {v}}^{T}_{l} \end{aligned}$$

where ${\mathbf {U}}$ is a singular left $J \times L$ matrix, ${\mathbf {V}}$ is a $K \times L$ is the singular right matrix and, $\Lambda$ is a $L \times L$ diagonal matrix of the *L* singular values. The key concept here is that the SVD provides the best reconstitution (in a least squares sense) of the original matrix by a matrix with a lower rank (Takane 2003).

PLS Correlation generalizes this idea to two tables. Given two variables *X* and *Y* the correlation matrix ${\mathbf {R}}$ is given by:

2 $$\begin{aligned} {\mathbf {R}} = {\mathbf {Z}}^{T}_{X}{\mathbf {Z}}_{Y} = {\mathbf {U}} \Lambda {\mathbf {V}}^{T} = \sum _{l}^{L}\delta _{l}{{\textbf {u}}}_{l}{{\textbf {v}}}^{T}_{l} \end{aligned}$$

where ${{\textbf {Z}}}_{X}$ and ${{\textbf {Z}}}_{Y}$ are the standardized matrices of observations, and the Latent Variables are denoted as the projection of the original matrices to the relative singular matrices:

3 $$\begin{aligned} {\mathbf {L}}_{X} = {\mathbf {Z}}_{X}{\mathbf {V}} \qquad {\mathbf {L}}_{Y} = {\mathbf {Z}}_{Y}{\mathbf {U}} \end{aligned}$$

The goal of PLSC is to find the latent vectors with maximal variance:

4 $$\begin{aligned} {\mathbf {l}}_{X,l} = {\mathbf {Z}}_{X}{\mathbf {v}}_{l} \quad \text {and} \quad {\mathbf {l}}_{Y,l} = {\mathbf {Z}}_{Y}{{\textbf {u}}}_{l} : \quad \text {cov}({\mathbf {l}}_{Y,l}, {\mathbf {l}}_{X,l}) \propto {\mathbf {l}}^{T}_{Y,l}{\mathbf {l}}_{X,l} = \text {max} \end{aligned}$$

whilst the goal of PLS Regression is to predict a table of variables using the information in another table of variables. PLSR aims to find a latent variable matrix *L* that *simultaneously* models ${\mathbf {X}}$ and predicts ${\mathbf {Y}}$

5 $$\begin{aligned} {\mathbf {X}}\;=\; & {} {\mathbf {T}}{\mathbf {P}}^{T} \quad \text {and} \quad \hat{{\mathbf {Y}}} = {\mathbf {T}}{\mathbf {B}}{\mathbf {C}}^{T} \end{aligned}$$

6 $$\begin{aligned} \hat{{\mathbf {Y}}}\;=\; & {} {\mathbf {X}}{\mathbf {B}}_{\text {PLS}} \nonumber \\ {\mathbf {B}}_{\text {PLS}}= & {} {\mathbf {P}}^{T}+{\mathbf {B}}{\mathbf {C}}^{T} \end{aligned}$$

where ${\mathbf {P}}$ and ${\mathbf {C}}$ are the *loadings* of ${\mathbf {X}}$ and ${\mathbf {Y}}$ respectively, and ${\mathbf {B}}$ is a diagonal matrix. Latent variables are ordered according to the variance of $\hat{{\mathbf {Y}}}$ that they explain. Equation 6 shows the regression form of previous equation, ${\mathbf {B}}_{\text {PLS}}$ is the multiple regression weights (loadings) with *J* rows and *K* columns.

PLS Path Modeling can be considered as the least square alternative to Structural Equation Modeling, which is based on maximum likelihood. Formally, a PLS-PM is defined as two sets of linear equations, the *inner* and *outer model*.

**Fig. 1:**
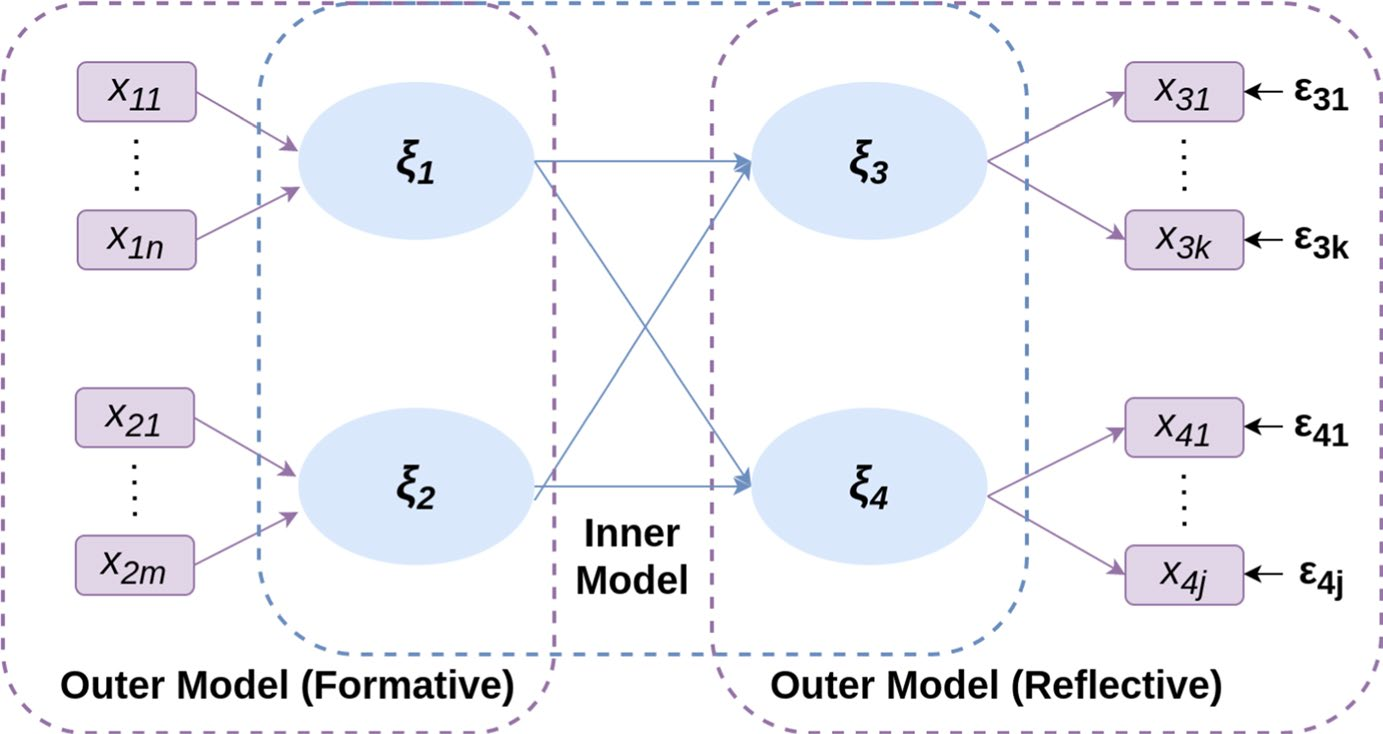
Path model definition

In Fig. 1, the *inner model* describes the relationships among the latent variables. The inner model is a causal chain system (i.e. with uncorrelated residuals and without correlations between the residual terms of a certain endogenous latent variable and its explanatory latent variables). Latent variables are the model measurements that are not directly observed, while manifest variables are measurements of the model directly observable. These relationships are represented in the following equation

7 $$\begin{aligned} \xi = {\mathcal {B}}\xi +\epsilon \end{aligned}$$

where $\xi$ is a vector of latent variables, ${\mathcal {B}}$ denotes the coefficient matrix of their relationships, and $\epsilon$ represents the inner model residuals. The assumption is that the inner model constitutes a causal chain system, the specification of predictors reduces the Eqs. (7) to (8).

8 $$\begin{aligned} (\xi \mid \xi ) = {\mathcal {B}}\xi \end{aligned}$$

The *outer model* describes the relationships between latent and manifest variables. Manifest variables are represented in two modes: i) Reflective (Mode A) and ii) Informative (Mode B). The reflective model has causal relationships from the latent variable to the manifest variables in its block. Thus, each manifest variable is assumed to be generated as a linear function of its latent variables and the residuals.

The formative mode of a measurement model has causal relationships from the manifest variables to the latent variable.

9 $$\begin{aligned} \begin{aligned} {\mathcal {X}}_{x}&= \Lambda _{x}\xi + \epsilon _{x} \\ \xi&= \Pi _{x}{\mathcal {X}}_{x} + \epsilon _{x} \end{aligned} \end{aligned}$$

Manifest variables are subject to predictors specification, which reduces Eqs. (9)–(10).

10 $$\begin{aligned} \begin{aligned} ({\mathcal {X}}_{x}\mid \xi )\;=\;&\Lambda _{x}\xi \\ (\xi \mid {\mathcal {X}}_{x})\;=\;&\Pi _{x}\xi \end{aligned} \end{aligned}$$

The PLS algorithm is essentially a sequence of regressions in terms of weight vectors. The weight vectors obtained at convergence satisfy fixed point Equations (Dijkstra 2010). The basic PLS algorithm includes the following three stages (Lohmöller 1989):

1. Iterative estimation of latent variable scores
  - Outer approximation of the latent variable scores (Tenenhaus et al. 2005)
  - Estimation of the inner weights
  - Inner approximation of the latent variable scores
  - Estimation of the outer weights
2. Estimation of outer weights/loading and path coefficients.
3. Estimation of location parameters.

These three steps are repeated until the difference in outer weights between two iterations is less than a predetermined threshold. After step one, the algorithm returns latent variable scores for all latent variables.

## Methodology

The method TOBIAS combines NLP techniques such as Sentiment analysis and Topic Modeling with PLS-PM to build an interpretable model, aiming to leverage the information expressed in the textual form to infer and explain user’s quality assessments and, definitely, support the quality assurance in improving the the overall quality of public services. Its algorithm is made up of four main steps:

1. Emotional Features extraction
2. Topics assignment
3. Overall quality indexes definition
4. PLS-PM model estimation

### Notation

Let us consider a collection of *N* documents ${\mathcal {D}} = \{d_1, \dots , d_N \}$, where each one is referred to a single course *c* taught by a lecturer *p* indicated by the pair element $(c,p) \in {\mathcal {O}}$. Specifically, each document *d* is considered as a collection of unordered sentences, that is $d = \{ s_1, s_2, \dots \}$, that are characterized by emotional features and an abstract topic that occurs in each of them. The collection of all sentences of the *N* documents is represented by the set ${\mathcal {S}} = \bigcup _{i = 1}^N \{\forall s \in d_i \}$, with a cardinality, that is the number of elements, $|{\mathcal {S}} |= n_{\mathcal {S}}$. According to the course and lecturer to which the sentence is referred, the set ${\mathcal {S}}$ can be partitioned into $|{\mathcal {O}} |$ subsets ${\mathcal {S}}_i = \{\forall s \in {\mathcal {S}} : \gamma (s) = (p,c)_i \}$ with $\gamma : {\mathcal {S}} \rightarrow {\mathcal {O}}$ is the function that maps the sentences to the corresponding course and lecturer (*p*, *c*) considered in them.

The first step of the algorithm consists in emotional features extraction. Let us consider a set of manifest emotional features ${\mathcal {F}} \in [0,n_{\mathcal {S}}]$ that can be measured from text that reflect a latent variable $\xi _{\mathcal {A}}$ representing the *Affectiveness*. According to the emotional states, these manifest variables ${\mathcal {F}}$ can be grouped into *K* subblocks $A_k$.

The second step of TOBIAS is the topics’ assignment. Let us define ${\mathcal {W}}$ as the collection of all words used in all sentences, that is the Bag-of-Words (BoW) of ${\mathcal {S}}$, and $\Theta = \{ \theta _1, \dots , \theta _H \}$ the collection of the topics occurred in ${\mathcal {S}}$. Topic modelling method $\phi (\cdot )$ maps the words of the BoW to topics such that $\phi : {\mathcal {W}} \rightarrow \Theta$. For each topic $\theta _h$ detected a latent variable $\xi _{\theta _h}$, which is expressed by a single manifest variable ${\mathcal {F}}_{\theta _h}$, is defined.

The third step consists in determining the manifest indexes expressing the latent overall quality of the services $\xi ^\star$ (called *Satisfaction*) as perceived by final users.

Next, the values referred to the same pairs (*p*, *c*) are aggregated modifying the number of the observations from $n_{{\mathcal {S}}}$ to the cardinality of ${\mathcal {O}}$. Finally, the fourth step concerns the setting of the PLS-PM framework of the formal model and its operative version, that is the model that fits the data.

### Formal model definition

**Fig. 2:**
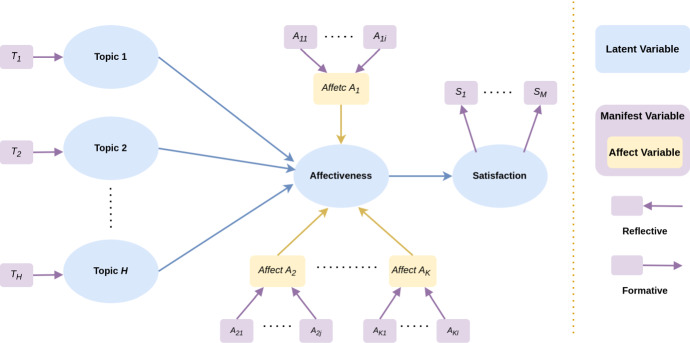
TOBIAS framework. Blue nodes represent the latent variables, violet nodes represent manifest variables, *reflective* are drawn using an arrow from the latent variable to the manifest variable while *formative* manifest variable are drawn with an arrow from the manifest to latent variable. Affective manifest variables are highlighted using yellow nodes. We consider *H* Topics, *K* affect measurements, and *M* satisfaction measurements

The framework of the formal model of TOBIAS is illustrated in Fig. 2. The inner model depicts the relation of *Satisfaction* depending on the *Affectiveness*, whilst the *H* latent variables $\xi _{\theta _h}$ *Topic* are modeled as the main drivers that trigger the *Affectiveness*.

Regarding the outer model, the *Satisfaction* block $\xi ^\star$ is reflective of *M* manifest variables expressing the overall quality of the services as perceived by final users. The *Topic* blocks $\xi _{\theta _1}, \dots , \xi _{\theta _H}$ are formative. Finally, *Affectiveness* is a formative block referring to all measures of the emotional state that can be measured from text, such as sentiment, emotion, etc. Its manifest variables can be grouped into *K* homogeneous sub-blocks *A* according to the emotional state they describe.

## Motivating example

### The case study

The spread of Coronavirus disease, which shattered the world in 2019, profoundly changed our daily life habits. Universities worldwide have had to quickly adapt to a remote mode for teachings and exams. The change in the way of teaching has entailed a need of furthered investigation on students’ satisfaction to understand the effects of the new elements introduced. Generally, the data on student satisfaction are collected through standardized Quality Assurance (QA) questionnaire, and the final output corresponds to quantitative indexes that are comparable between courses. Studying the specific reasons that produced the values of those quantitative indexes is difficult since the possible causes that could influence the overall satisfaction of the students vary between courses, and, at the same time, it is not possible to customize the questionnaires on the courses too much otherwise, there would be a risk of losing comparability. Nonetheless, an essential source of information that can be considered to overcome this problem is the open-ended question, usually set at the end of the questionnaire, that gives to the students the opportunity to express their issues. Since its answer consists of a text, often it is read by the analyst without being used in the analysis.

In this section, we present a case of study of the University of Cagliari during the first two years of the COVID-19 pandemic (2020-21), in which we carry out TOBIAS to model and infer students’ overall satisfaction assessments by using the information extracted from students’ issues reports.

The QA questionnaire filled by the students of the University of Cagliari is organized in three main areas of interest: i) course subject; ii) teaching; iii) interest and satisfaction. In addition to the open-ended question where the student can report their issues about the teacher and the course, the latter contains two questions that concern the overall satisfaction: i) the overall satisfaction with the subject of the course; ii) the overall satisfaction of the student with the teaching. All the closed questions of the QA questionnaire have an ordinal scale with four responses: (i) “NO”; (ii) “more NO than YES”; (iii) “more YES than NO”; (iv) “YES”.

To elaborate on the results, the analysts of the University of Cagliari use the ordinal responses frequencies to evaluate the following QA indexes defined in Eqs. (12) and (11): Positiveness Index (PI), and Strength Index (SI).

11 $$\begin{aligned} \text {PI}= & {} \frac{\# {``YES''} + \#{``more YES than NO''}}{\#\text {total answers}} \end{aligned}$$

12 $$\begin{aligned} \text {SI}= & {} \left\{ \begin{array}{ll} \frac{\# \hbox {``YES''} }{\# \hbox {``YES''} + \#\hbox {``more YES than NO''}} &{} \text { if PI} > 0.5\\ \\ \frac{\#\hbox {``more NO than YES''} }{\# \hbox {``NO''} + \#\hbox {``more NO than YES''} } &{} \text { if PI} \leqslant 0.5 \end{array}\right. \end{aligned}$$

**Fig. 3:**
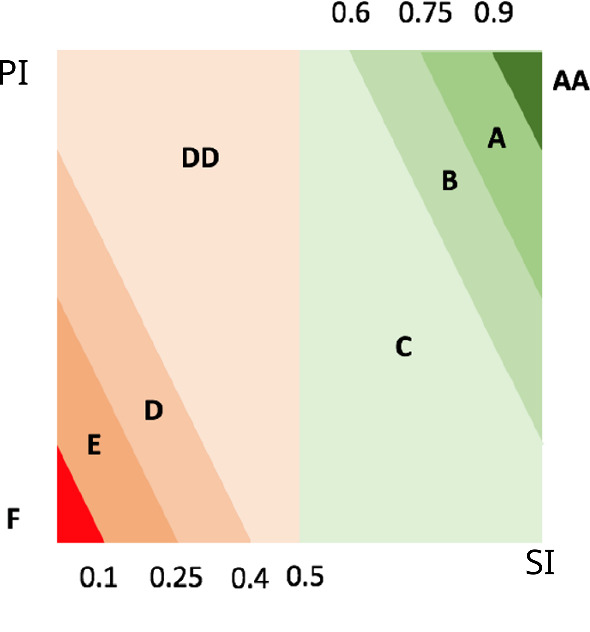
Graphical representation of Assessments evaluation classes

The Positiveness Index (PI) measures the proportion of positive answers over the total ones. The Strength Index (SI) instead, measures the intensity of the positive/negative polarity of the answers. By combining the indexes PI and SI as illustrated in Table 1, the analysts produce eight ordinal evaluation classes, that can be depicted in a Cartesian plane (Fig. 3).

**Table 1:** Assessments evaluation classes definition

Class	*PI*	*SI*	
AA	${PI} > 0.90$	$-2\cdot PI + 2.8 < {SI}$	$\leqslant 1$
A	${PI} > 0.75$	$-2\cdot PI + 2.5 < {SI}$	$\leqslant -2\cdot PI + 2.8$
B	${PI} > 0.60$	$-2\cdot PI + 2.2 < {SI}$	$\leqslant -2\cdot PI + 2.5$
C	${PI} > 0.50$	$0 \leqslant {SI}$	$\leqslant -2\cdot PI + 2.2$
DD	${PI} \leqslant 0.50$	$-2\cdot PI + 0.8 < {SI}$	$\leqslant 1$
D	${PI} \leqslant 0.40$	$-2\cdot PI + 0.5 < {SI}$	$\leqslant -2\cdot PI + 0.8$
E	${PI} \leqslant 0.25$	$-2\cdot PI + 0.2 < {SI}$	$\leqslant -2\cdot PI + 0.5$
F	${PI} \leqslant 0.10$	$0 \leqslant {SI}$	$\leqslant -2\cdot PI + 0.2$

### Features definition

In this study, we analyzed $N=1485$ students reports containing $n_{\mathcal {S}} = 3871$ sentences, which refer to the academic years 2019-20 and 2020-21 (Table 2).

**Table 2:** Dataset statistics

Dataset statistics	
Students’ reports	1485
Sentences	3871
Courses	649
Lecturers	515
Academic years	2

In the Emotional Features extraction step, $K = 3$ subblocks of emotional states have been considered, specifically *Sentiment* ($A_S$), *Emotion* ($A_E$), and *Moods* ($A_M$). Both *Sentiment* and *Emotion* are made up of a single manifest variable, respectively, ${\mathcal {F}}_{\text {Sen}}$ and ${\mathcal {F}}_{\text {Emo}}$. They are measured by using the classifier UmBERTo which has been specifically designed for Italian language and trained on FEEL-IT dataset (Bianchi et al. 2021). In particular, for ${\mathcal {F}}_{\text {Sen}}$ UmBERTo maps sentences into positive or negative sentiment that is $U_1: {\mathcal {S}} \rightarrow \{\text {pos},\text {neg}\}$. Instead, for ${\mathcal {F}}_{\text {Emo}}$ UmBERTo identifies the most relevant emotion of the sentence in such a way $U_2: {\mathcal {S}} \rightarrow \{\text {joy}, \text {anger}, \text {fear}, \text {sadness}\}$. Then their values are averaged by the (*p*, *c*) elements associated. Specifically, considering the subset ${\mathcal {S}}_i$, their *i*-th values are the following

13 $$\begin{aligned} \begin{aligned} {\mathcal {F}}_{\text {Sen},i}&= \sum _{s \in {\mathcal {S}}_i} \frac{\left[ I\Big (U_1(s) \in \{\text {pos}\}\Big ) - I\Big (U_1(s) \in \{\text {neg}\}\Big ) \right] }{|{\mathcal {S}}_i |} \\ {\mathcal {F}}_{\text {Emo},i}&= \sum _{s \in {\mathcal {S}}_i} \frac{\left[ I\Big (U_2(s) \in \{\text {joy}\}\Big ) - I\Big (U_2(s) \in \{\text {anger},\text {fear},\text {sadness}\}\Big ) \right] }{|{\mathcal {S}}_i |} \end{aligned} \end{aligned}$$

where $I(\cdot )$ is an indicator function that is equal to one if the condition is respected otherwise to zero, and $|{\mathcal {S}}_i |$ indicates the cardinality of ${\mathcal {S}}_i$.

In the subblock $A_M$, five moods and as much features have been considered: *worried* (${\mathcal {F}}_{\text {Wor}}$), *sad* (${\mathcal {F}}_{\text {Sad}}$), *amused* (${\mathcal {F}}_{\text {Amu}}$), *satisfied* (${\mathcal {F}}_{\text {Sat}}$) and *indignant* (${\mathcal {F}}_{\text {Ind}}$). The features are computed by using the function $\text {spaCy}(\cdot )$ (SpaCy 2022) trained on the emotion lexicon called DEPECHE-MOOD++ (Staiano and Guerini 2014). spaCy computes the normalized weights of the moods of each sentence, such that $\text {spaCy}: {\mathcal {S}} \rightarrow [0,1]^5$. Considering $\left[ \text {spaCy}(s)\right] _m$ as the obtained normalized weight of the mood *m*, their *i*-th values are computed as follows

14 $$\begin{aligned} {\mathcal {F}}_{m,i} = \sum _{s \in {\mathcal {S}}_i} \left[ \text {spaCy}(s)\right] _{m} \quad \text { with } m = \text {Wor}, \text {Sad}, \text {Amu}, \text {Sat}, \text {Ind}. \end{aligned}$$

Regarding the topics, by using seeded LDA we identified: $H = 4$ topics: *Exam*, *Slides*, *Teacher* and *Labs*. Table 3 shows the *initial seed words* provided to the Seeded-LDA along with the top ten most representative keywords for each topic. We empirically defined the number of topics equals to four, by manually analysing students’ issues, and for the sake of interpretability of the topics we decided that four topics were the best trade-off. The first topic concerns the final exam, the type of evaluation, and the presence of middle-term exams. The second topic concerns the study and teaching materials such as slides, the official book and the online settings which were due to the Covid pandemic. The third topic concerns the teacher itself, her/his availability, the teaching skills, and the overall preparation. The last topic concerns the auxiliary teaching activities such as: labs, exercise with the tutor, and the evaluation of the tutor itself.

**Table 3:** Topics interpretation

Label	Description	Seed words	Top keywords
Exam	Comments on exam rules, partial exams etc.	Appello (*time round*), esame (*exam*), orale (*oral*), scritto (*written*), intermedio (*middle-term*), valutazione (*evaluation*), modalità (*mode*), domanda (*question*), modalità esame (*examination methods*)	Lezione (*lesson*), studente (*student*), docente (*teacher*), chiaro (*clear*), spiegazione (*explanation*), corso (*course*), disponibile rispondere (*available answer*)
Slides	Comments on Slides, Teaching Material, PC etc.	Materiale didattico (*teaching material*), testo (*textbook*), slides , attività (*activity*), libro (*book*), registrazione (*registration*), online, pc, computer, didattica (*teaching*)	Materia (*subject*), insegnamento (*teaching course*), studio (*study*), fornire (*to provide*), seguire (*to attend*), spiegazione (*expanation*)
Teacher	Comments on Teacher competence, availability and punctuality	Docente (*teacher*), professore (*professor*), professoressa (*professor*), prof, prof.ssa	Disponibile (*available*), spiegare (*to explain*), insegnamento (*teaching*), svolgere (*to carry out*), studiare (*to study*), conoscenza (*knowledge*), concetto (*concept*)
Labs	Comments on Labs, Exercises and Tutoring	Tutor, laboratorio (*laboratory*), tirocinio (*internship*), tutoraggio (*tutoring*), esercizi (*exercise*), organizzazione (*organizer*), assistente (*assistent*), esercitazione (*practice exercise*)	Tutoraggio (*tutorship*), rendere chiaro (*to make clear*), spiegazione (*explanation*), prova (*trial*), preparazione (*preparation*)

The first column represents the topic’s label. The second column reports the topic’s interpretation. The third column lists the initial seed words provided to Seeded-LDA. Finally, the last column shows the words within the top ten most representative keywords for each topic beyond the seed words provided. In the last two columns the original Italian words are in regular whilst in italics and in brackets the corresponding translation into English when needed

Each latent variable is characterized by a single manifest variable. Considering $p({\theta _h} |s)$ as the posterior probability of the topic $\theta _h$ in a sentence *s* provided by the LDA, the *i*-th values of the manifest variables of the topics are computed as follows

15 $$\begin{aligned} {\mathcal {F}}_{\theta _h,i} = \sum _{s \in {\mathcal {S}}_i} I\left( {{\,\mathrm{argmax}\,}}_{j = 1,\dots ,4} p({\theta _j} |s) = h \right) . \end{aligned}$$

Finally, for the latent variable $\xi ^\star$ two features are considered. The first one is simply the averages of the scores of the index IC. Specifically, its *i*-th value is

16 $$\begin{aligned} {\mathcal {F}}_{\text {ic},i} = \frac{\sum _{s \in {\mathcal {S}}_i} \text {IC}(s)}{|{\mathcal {S}}_i |}. \end{aligned}$$

The second feature ${\mathcal {F}}_{\text {class}}$, instead, is generated from the Assessments evaluation classes. We defined a function $z(\cdot ) : {\mathcal {S}} \rightarrow \{-2,-1,0,1,2,3\}$, that specifically maps the sentences with a class AA to 3, with A to 2, with B to 1, with C to 0, with DD to $-1$ and with D, E or F to $-2$. Then the *i*-th value of ${\mathcal {F}}_{\text {class}}$ is computed as follows

17 $$\begin{aligned} {\mathcal {F}}_{\text {class},i} = \frac{\sum _{s \in {\mathcal {S}}_i} z(s)}{|{\mathcal {S}}_i |}. \end{aligned}$$

### Exploratory analysis of features

**Fig. 4:**
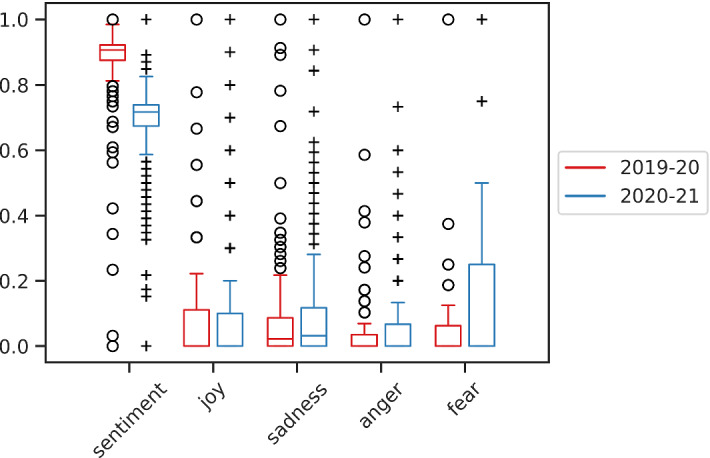
Normalized version of Sentiment ${\mathcal {F}}_{\text {Sen}}$ and the four emotions in the two academic years analyzed

In order to study the relationship between the features previously defined, we performed an exploratory analysis. Figure 4 shows the normalized version of ${\mathcal {F}}_{\text {Sen}}$ (*sentiment*) and those of the four emotions, as defined by the function $U_2(\cdot )$ of UmBERTo, during the two years considered. As it can be noted, there is a general decrease of positive sentiment and emotion and a complementary increase of negative ones from 2019-20 to 2020-21.

**Fig. 5:**
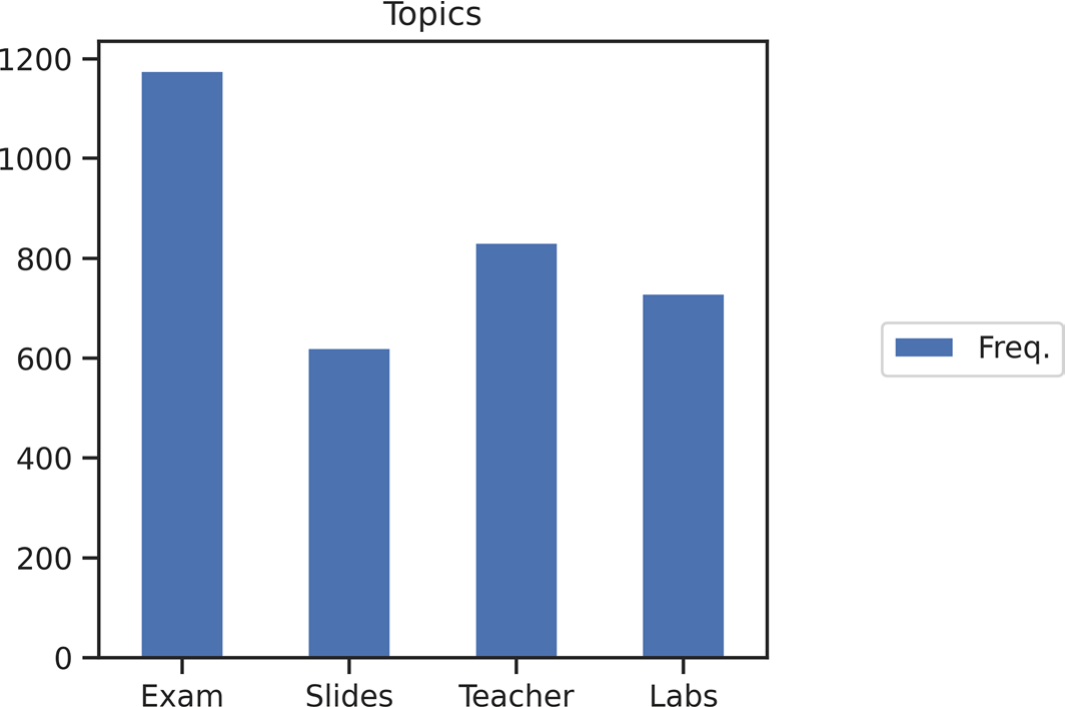
The distribution of the prevalent topic of the all sentences

The distribution of the prevalent topic of the 3871 sentences is showed in Fig. 5. The topic *Exam* is the one with the highest frequency, while the other three have similar occurrences.

**Fig. 6:**
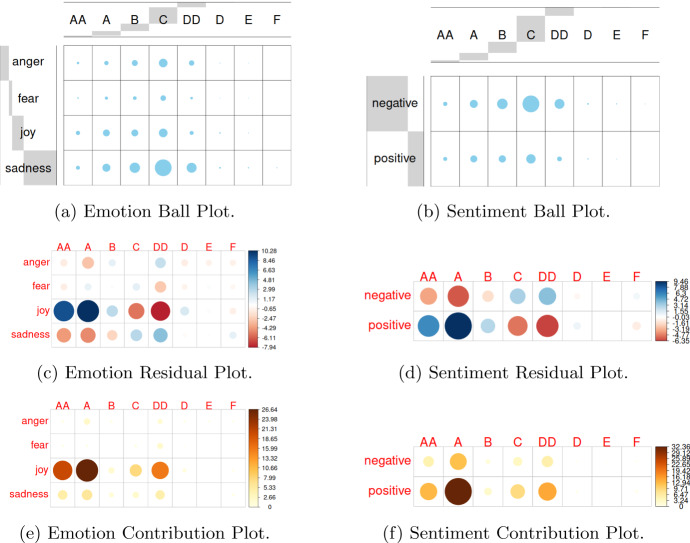
Stochastic independence analysis of emotions and sentiments versus evaluation Classes

By considering the relative contingencies tables, we analysed the degree of association between the two sentiments (either positive or negative) and the four emotions, as defined respectively by the functions $U_1(\cdot )$ and $U_2(\cdot )$ of UmBERTo, and the Evaluation classes. Figure 6a and b show the contingency tables of the two observed categories: Emotions and Evaluation Class, Sentiments and Evaluation Class. This figure shows a graphical matrix where each cell contains a dot whose size reflects the relative magnitude of the corresponding joint frequency. We can see that the observed joint frequencies (Emotion/Sentiment, Evaluation class) tend to be distributed on specific (Emotion/Sentiment, Evaluation class) cells, indicating a stochastic dependence.

Furthermore, we tested their independence by Chi-Square test, obtaining that they are significantly associated ($p < 0.001$). The degree of association explained by each cell can be calculated using the Chi-square residual statistic for each cell the so-called Pearson residuals (Sharpe 2015). Figure 6c and d show the Pearson residuals, where circles size are proportional to the cell contribution. Here it is very important the sign of the standardized residuals, which is crucial to interpret the relationship between rows (Sentiment/Emotion) and columns (Evaluation class). The sign of the standardized residual is interpreted as follows: (i) Positive residuals are highlighted in blue and indicate a positive association (attraction) between the relevant row and column variables; ii) Negative residuals are shown in red and suggest that the associated row and column variables have a repulsion (negative relationship).

The contribution of each cell to the total Chi-square score is obtained by the following ratio: $contr = r^2 / \chi ^2$, where *r* is the cell Pearson residual. Figure 6e and f show these contributions. Considering the emotions, the major contributions are given by *joy*, *sadness*, where the former has a positive association with positive evaluations (AA and A) and negative association with negative evaluation (DD) and the latter has a negative association with positive scores (AA, A) and positive association with negative evaluation (DD). We can also note that *anger* emotion provides the highest contribution for the C evaluation, which is a slightly positive evaluation with some critical aspects and a wide range of improvement. The highest contribution are given by *joy* and *sadness* for the DD evaluation class (with opposite association).

**Fig. 7:**
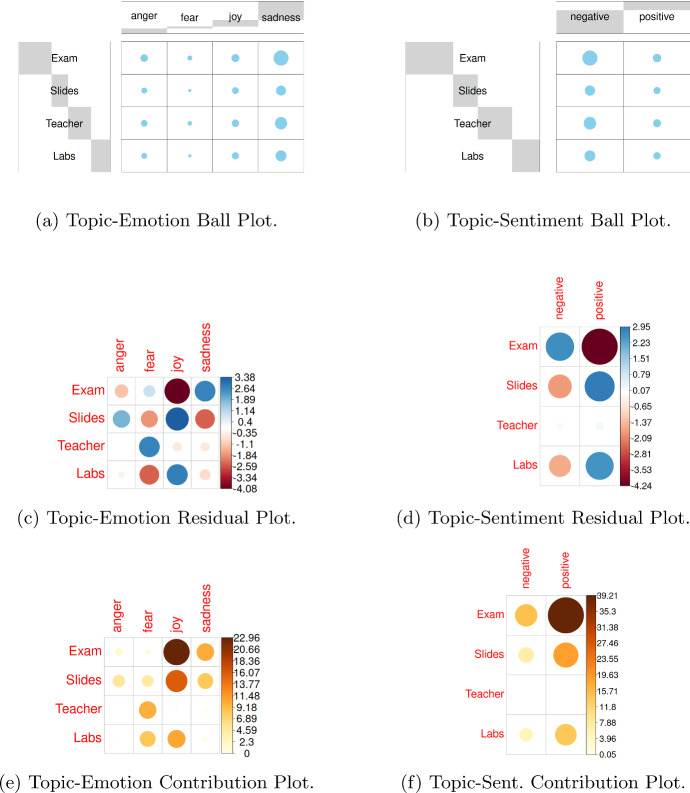
Stochastic independence analysis of topics versus emotions and sentiments

Figure 7 shows the relationship between topic and sentiment (emotion). We can see that the highest repulsion (negative association) is between the *Exam* topic and *positive* sentiment and *joy* emotion, while the highest attraction (positive association) is between *Exam* topic and *negative* sentiment as *sadness* emotion. There are other relationships, such as the attraction (positive association) between the topic *Teacher* and the emotion *fear*. Figures 6 and 7 highlight some relationships among topics discussed by students and the *affectiveness* expressed by students in their comments, and among the evaluation of students with respect to the teaching and their *affectiveness*. These relationships motivated us to apply our *TOBIAS* model to this study case, in order to better understand the underlying relationship among topic discussed, affectiveness expressed, and final quality assessment of students.

### TOBIAS model results

**Fig. 8:**
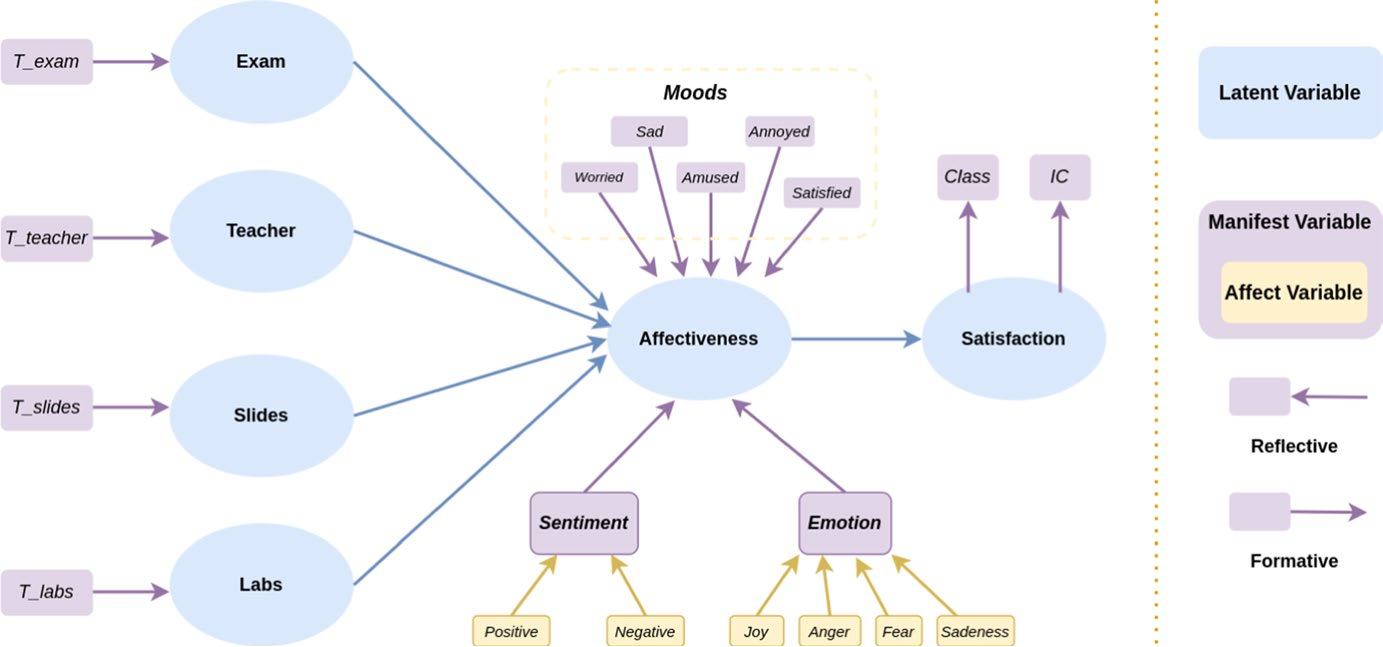
Path Model definition. Blue nodes represent the latent variables, violet nodes represent manifest variables, *reflective* are drawn using an arrow from the latent variable to the manifest variable while *formative* manifest variable are drawn with an arrow from the manifest to latent variable. Affective manifest variables are highlighted using yellow nodes

Figure 8 shows TOBIAS framework of our case study. We identified four topics, a dimension of affective with seven measurements, and two measurements of the satisfaction. Blue nodes represent the latent variables, violet nodes represent manifest variables. Reflective variables are drawn using an arrow from the latent variable to the manifest variable while formative manifest variable are drawn with an arrow from the manifest to latent variable. The variables used in the definition the manifest variables of Sentiment and Emotion are highlighted using yellow nodes.

**Fig. 9:**
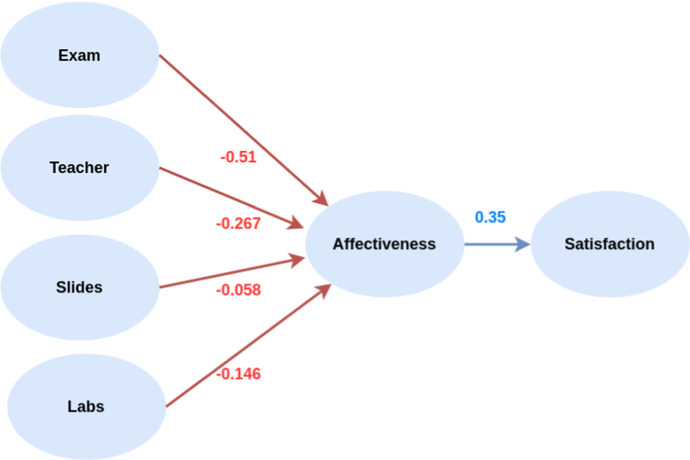
Inner model

By fitting TOBIAS model the results shown in Figs. 9 (the inner model) and 10 (the outer model) are obtained. The inner model shows a negative impact of the topics on the *Affectiveness*, which in turn has an overall positive influence to *Satisfaction*. We can also note that the highest impact of topics on affectiveness is provided by the topic *Exam* while the lowest negative impact is due to the *Slides*. The outer model shows a similar contribution of ${\mathcal {F}}_{\text {ic}}$ and ${\mathcal {F}}_{\text {class}}$ manifest variables to *Satisfaction*, while *Affectiveness* leads to interpretable insights. We can see that ${\mathcal {F}}_{\text {Sen}}$ and ${\mathcal {F}}_{\text {Emo}}$ have a positive influence on the *Affectiveness* which we recall has a positive effect on the *Satisfaction*. Positive moods such as ${\mathcal {F}}_{\text {Sat}}$ and ${\mathcal {F}}_{\text {Amu}}$ show a positive effect as well, while negative moods such as ${\mathcal {F}}_{\text {Sad}}$ and ${\mathcal {F}}_{\text {Ind}}$ exhibit a negative effect. The influence of the ${\mathcal {F}}_{\text {Wor}}$ is positive meaning that the more the students are worried the more the overall satisfaction evaluation is positive, this result may seem counter-intuitive at a first gist, but we should consider that mood, differently from emotion, are long-lasting feelings and that this case study is conducted during the two years of Covid-19 pandemic, thus seems reasonable that ubiquitous sense of worry.

**Fig. 10:**
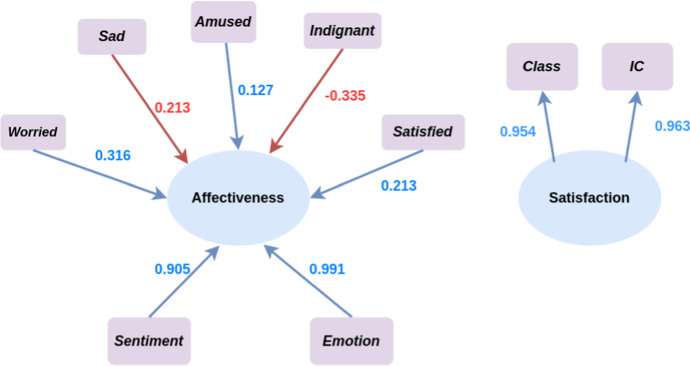
Outer model

The evaluation of the TOBIAS model is summarized in Tables 4 and 5. Table 4 shows its the structural assessment, showing the results of the regressions of each block of latent variables in the inner model. All latent variables are statistically significant except for *Slides* topic. We thus conclude that the comments of students concerning teaching material and slides are not significant (it also has the lower loading toward *Affectiveness* among all topics).

**Table 4:** Structural assessment of TOBIAS model

	Estimate	Std. Error	t value	Pr($>|t|$)
*Affectiveness*				
Intercept	0.00	0.0107	0.00	1.00
Exam	$-0.51$	0.0287	$-14.01$	***
Slides	$-0.06$	0.0326	$-1.45$	0.15
Teacher	$-0.27$	0.0346	$-6.30$	***
Labs	$-0.15$	0.0347	$-3.41$	***
*Satisfaction*				
Intercept	0.00	0.0355	0.00	1.00
Affectiveness	0.35	0.0355	9.81	***

Results of the regressions of Affectiveness and Satisfaction Blocks. The statistical significance regression of blocks are indicated as follows: *** $p< 0.001$, ** $p< 0.01$, * $p<0.05$

**Table 5:** Inner model summary

	Type	$R^2$	Block communality	Mean redundancy
Exam	Exogenous	0.00	1.00	0.00
Slides	Exogenous	0.00	1.00	0.00
Teacher	Exogenous	0.00	1.00	0.00
Labs	Exogenous	0.00	1.00	0.00
Affectiveness	Endogenous	0.90	0.30	0.27
Satisfaction	Endogenous	0.12	0.92	0.12

$R^2$ indicates the amount of variance in the endogenous latent variable explained by its independent latent variables. *Communalities* are squared loadings and they measure the part of the variance between a latent variable and its indicator that is common to both. *Redundancy* is the amount of variance in an endogenous construct explained by its independent latent variables

Besides the results of the regression equations, the quality of the structural model is evaluated by examining three indices or quality metrics: the $R^2$ determination coefficients, the block Communality and the Mean Redundancy. Table 5 shows these metrics for all latent variables.

The $R^2$ indicates the amount of variance in the endogenous latent variable explained by its independent latent variables. TOBIAS model explains 90% of the total variance of *Affectiveness* with the four topics. The total variance of the *Satisfaction* explained by *Affectiveness* is 12%.

The Communality is a measure of how well a block is explained by its indicators (manifest variables). Here *Satisfaction* has a high ($>90\%$) meaning that the latent variables is well explained by their indicators.

The redundancy index reflects the ability of a set of independent latent variables to explain variation in the dependent latent variable. In our case study the redundancy index confirms the interpretation of the $R^2$.

## Conclusions

This study proposes a methodology to model the effects of the topics, moods, and sentiments expressed in service users’ comments describing a phenomenon, upon its overall rating. We presented the TOpic Based Index Assessment through Sentiment framework. TOBIAS is constructed by combining different natural language processing techniques and statistical methodologies. The first step is the Sentiment Analysis which identifies sentiments, emotions, and moods, and Topic Modeling analysis which finds the main relevant topics discussed in the comments. In the next step, the Partial Least Square with Path Modelling estimates how they affect an overall rating that summarizes the performance of the analysed phenomenon.

The new methodologies have been applied to the text of the comments written by the University of Cagliari (Italy) students to evaluate the lecturers and courses. In addition, we conducted a case study on the student evaluation of the teaching quality, and we applied the TOBIAS framework. We conducted our case study during the last two years of the COVID-19 pandemic. The spread of Coronavirus disease, which shattered the world in 2019, profoundly changed our daily life habits. Universities worldwide have had to quickly adapt to a remote mode of teaching and exams.

TOBIAS model highlighted the negative impact of the topic discussed by the student on the overall sentiment, emotion, and moods which on the contrary positively influence on the overall satisfaction expressed on the teaching. These results were supported by the explorative analysis and extended in interpreting the TOBIAS model. The preliminary analyses showed the highest impact of the Topic *Exam* on sentiments and emotions, and with the TOBIAS model we found that the highest negative contribution to *Affectiveness* is given by the *Exam* latent variable. In other words, thanks to TOBIAS we have been able to understand better the existing relationships between the topics discussed and the affectiveness expressed by students in their comments, which are not identifiable through solely an exploratory analysis. Although the results obtained are encouraging, our approach poses some limitations. The dimensions of Affectiveness discussed in the motivating example showed some correlation, for example between Emotion and Moods. These correlations may impact the final output of the model and will be considered in future works.
